# Theoretical Analysis of the Refractometric Sensitivity of a Coated Whispering Gallery Mode Resonator for Gas Sensing Applications

**DOI:** 10.3390/s22239155

**Published:** 2022-11-25

**Authors:** Davor Ristić, Daniil Zhivotkov, Snigdha Thekke Thalakkal, Elena Romanova, Mile Ivanda

**Affiliations:** 1 Ruđer Bošković Institute, Bijenička Cesta 54, 10000 Zagreb, Croatia; 2 Institute of Physics, Saratov State University, Astrakhanskaya Ulitsa, 83, 410012 Saratov, Russia

**Keywords:** whispering gallery modes, refractometric sensing, microspheres

## Abstract

We present a theoretical analysis of the refractometric sensitivity of a spherical microresonator coated with a porous sensing layer performed for different whispering gallery modes. The effective refractive index of the modes is also calculated. The calculations are also made for a system which has an additional high-refractive index layer sandwiched between the microsphere and the porous sensing layer. The results of the calculation are discussed in regards to the applicability of the studied systems for gas sensor construction.

## 1. Introduction

Whispering gallery mode (WGM) resonators are very high Q-factor, low modal volume optical resonators which have recently attracted a lot of interest in the scientific community. Their high quality makes them very interesting for a number of applications [1] such as lasing [2], frequency comb generation [3,4] and sensing. Sensing, in particular, has been a particularly fruitful application of WGM resonators since 2002, when the first bio-sensor based on whispering gallery modes was fabricated [5]. Since then, a number of papers on bio-sensing using WGM resonators have been reported [6,7,8]. The principle of bio-sensing using WGMs of a spherical resonator is to track the shift of the eigenfrequency of a particular resonator induced by the binding of an analyte molecule onto the sphere surface. The basic sensing principle is refractometric: the analyte induces a change of the refractive index of the resonator leading to the change of the optical path of the photon circulating in the microresonator which in turn induces a change of the mode eigenfrequency. The drawback of this method is that, when the analyte bonds to the surface, it can only interact with a very small portion of the electric field of the WGM. The WGM is confined inside the resonator by total internal reflection, making the electric field outside of the resonator an exponentially decaying evanescent tail. This severely limits the refractometric sensitivity, although this is somewhat offset by the fact that the high quality of the resonators makes possible the tracking of very small shifts of the WGM frequency. Recently, a new approach to sensing using WGM microresonators has been proposed which is particularly suitable for gas sensing [9,10,11,12,13,14]. When a microresonator is coated with a thick porous layer, the electric field of the WGM can be engineered to be confined inside the coating. The porosity of the coating enables the molecules of the analyte to penetrate inside the coating where they can interact with the WGM electric field, which is much stronger inside the coating than on the surface of the sphere. This can greatly increase the refractometric sensitivity. In 2017, Mallik et al. successfully used an agarose-coated microsphere to detect water vapors [9]. The same group also managed to detect ammonia vapors using a porous silica-coated microsphere [10,11,12]. More recently, Zhivotkov et al. [13,14] managed to increase the refractometric sensitivity of the porous silica sol-gel-coated microsphere to more than 1000 nm/RIU, leading to ammonia detection sensitivity of more than 100 nm/ppm while retaining very high Q-factor (between Q = 10^5^–10^6^). This value of 1000 nm/RIU is among the highest available in literature so far for resonator-based refractometric sensors. If a microsphere without any coating is used for sensing using the evanescent tail of the WGM at the surface of the sphere, the refractometric sensitivity depends on the sphere diameter and is between 13 and 2 nm/RIU for sphere diameters between 60 and 300 μm. Microbubble resonators with very thin walls have been recently used to achieve refractometric sensitivities between 18.8 nm/RIU with Q = 2.9 × 10^6^ [15] and 256.2 nm/RIU with Q = 4.67 × 10^4^ [16]. Microring resonators have been produced achieving sensitivities of 70 nm/RIU with Q = 2 × 10^5^ [17] and 212 nm/RIU with Q = 1.8 × 10^3^ [18]. The results reported in Zhivotkov et al. [13,14] show higher sensitivity than microbubble, microring and uncoated microspheres while still retaining high Q-factors. However, a detailed theoretical study on the refractometric sensitivity of a microsphere coated with a porous material is still lacking in literature. The theory of eigenfrequencies of a coated microsphere has already been studied for use in modal dispersion compensation [19,20], thermo-optic coefficient tailoring [21] and refractometric sensing using the evanescent field of the surface of the microresonator [22,23,24]. In this paper we present the theoretical analysis of the refractometric sensitivity of a system which consists of a microsphere coated with a porous layer and of a microsphere coated with two layers: one high refractive index layer that is used for electric field profile tailoring, and a porous layer that serves as the sensing medium.

## 2. Materials and Methods

**Figure 1:**
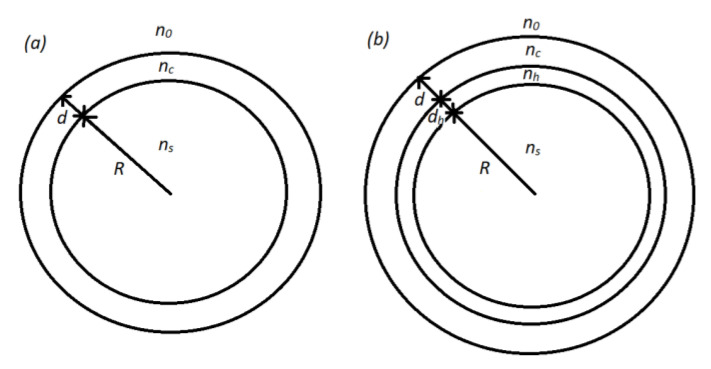
The sketch of a microsphere coated with a single layer (**a**) and with two layers (**b**).

The overall sensitivity of any refractometric sensor can be separated into two parts:

(1) $$\frac{\partial \lambda }{\partial c}=\frac{\partial \lambda }{\partial n}\frac{\partial n}{\partial c}\;$$

where *c* is the concentration of the analyte, *n* is the refractive index of the sensing medium and λ is the wavelength of a resonator mode. In Equation (1), ∂n/∂c is a property of the chemical interaction between the analyte and the material the resonator is made of, and in general cannot be tailored short of changing the chemistry of the materials in question. On the other hand, ∂λ/∂n is a property of the resonator geometry and can be tailored by changing the geometrical parameters of the resonator. To calculate ∂λ/∂n for a microresonator coated with a porous layer we consider the following two cases. The first system that we study is a microsphere with refractive index ns and radius *R* coated with a porous layer with refractive index *n_c_* and thickness d. The second system is a double-coated microsphere, which has an additional high-refractive index (*n_h_*) layer of thickness *d_h_* sandwiched between the sphere and the porous coating. In both cases the coated sphere is imbedded in a surrounding medium with refractive index *n*_0_. The two systems are sketched in Figure 1.

Spherical microresonators have mostly been used to detect analyte molecules which bond to the surface of the sphere [5,6,7,8]. When a molecule bonds to the surface, this can be viewed as a change of the surrounding medium the sphere is embedded in (*n*_0_). In that case, the corresponding refractometric sensitivity is equal to $\partial \lambda /\partial n_{0\;}$ [22,23,24]. In this paper, we are considering such a case when the analyte molecules can diffuse into the interior of the coating and change its refractive index (*n_c_*) so the corresponding refractometric sensitivity is $\partial \lambda /\partial n_{c}$. For this, we first need to calculate the mode eigenfrequencies of the two systems presented in Figure 1. The electric field $\vec{E}$ in our system has to satisfy the vector Helmholtz equation which can be solved using the Hansen method [25]. There exist two independent physical solution corresponding to two possible light polarizations:

(2) $$\vec{M}=\vec{\nabla }\phi \times \vec{r},\;\;\;\;\vec{N}=\frac{1}{nk}\vec{\nabla }\times \vec{M}$$

where ϕ is the solution to the scalar Helmholtz equation and is therefore equal to the product of a spherical Harmonic $Y_{lm}\left(\theta ,\varphi \right)$ and of a linear combination of spherical Bessel $j_{l}$ and Neumann $y_{l}$ functions $\psi_{l}=Aj_{l}\left(nkr\right)+By_{l}\left(nkr\right)$, where k is the wave propagation vector in vacuum and *n* is the refractive index of the material. Therefore, the two solutions $\vec{M}$ and $\vec{N}$ can be written in spherical coordinates as:

(3) $$\vec{M}=\psi_{l}\left(\frac{1}{\sin\theta }\frac{\partial Y_{lm}}{\partial \varphi }\hat{\theta }-\frac{\partial Y_{lm}}{\partial \theta }\hat{\varphi }\right)\;\vec{N}=\frac{l\left(l+1\right)}{nkr}\;\psi_{l}Y_{lm}\hat{r}-\frac{1}{nkr}\frac{\partial (r\psi_{l})}{\partial r}\left(\frac{\partial Y_{lm}}{\partial \theta }\hat{\theta }+\frac{1}{\sin\theta }\frac{\partial Y_{lm}}{\partial \varphi }\hat{\varphi }\right)$$

The electric and magnetic fields that satisfy the vector Helmholtz equation have to be constructed from vectors $\vec{M}$ and $\vec{N}$. The usual way is to define the TE (TM) modes as the modes for which the electric (magnetic) field is purely tangential to $\hat{r}$ [26]:

(4) $$\vec{E_{TE}}=E_{0}n\vec{M}\;,\;\vec{B_{TE}}=E_{0}\frac{in^{2}}{c}\vec{N}\;\vec{E_{TM}}=B_{0}\frac{1}{ic}\vec{N}\;,\;\vec{B_{TM}}=B_{0}n\vec{M}$$

where *c* is the velocity of light. The components of $\vec{E}$ and $\vec{B}$ perpendicular to $\hat{r}$ have to be continuous across all the boundaries between different materials. By looking at Equations (3) and (4) it can be seen that this condition is satisfied if $n\psi_{l}$ and $P\partial \left(r\psi_{l}\right)/\partial r$ are continuous across the boundaries where P=n for the TE and P=1/n for the TM mode. Since $\psi_{l}$ must not diverge anywhere in space, its general form in our particular case will be the Bessel function in the sphere core, a general linear combination of Bessel and Neumann function in the coatings, and the Neumann function in the surrounding medium. With two boundary conditions per each boundary, we will have in total four boundary conditions in the case of a single layer and six in the case of two layers. To find the eigenfrequencies we need to find the zeros of the determinants of the two boundary condition systems:

(5) $$\left|\begin{array}{cccc} S_{l}\left(kn_{s}R\right) & -S_{l}\left(kn_{c}R\right) & -C_{l}\left(kn_{c}R\right) & 0\\ P_{s}S_{l}{}^{\prime }\left(kn_{s}R\right) & -P_{c}S_{l}{}^{\prime }\left(kn_{c}R\right) & -P_{c}C_{l}{}^{\prime }\left(kn_{c}R\right) & 0\\ 0 & S_{l}\left(kn_{c}R_{c}\right) & C_{l}\left(kn_{c}R_{c}\right) & -C_{l}\left(kn_{0}R_{c}\right)\;\\ 0 & P_{c}S_{l}{}^{\prime }\left(kn_{c}R_{c}\right) & P_{c}C_{l}{}^{\prime }\left(kn_{c}R_{c}\right) & -P_{0}C_{l}{}^{\prime }\left(kn_{0}R_{c}\right) \end{array}\right|=0\;\left|\begin{array}{cccccc} S_{l}\left(kn_{s}R\right) & -S_{l}\left(kn_{h}R\right) & -C_{l}\left(kn_{h}R\right) & 0 & 0 & 0\\ P_{s}S_{l}{}^{\prime }\left(kn_{s}R\right) & -P_{h}S_{l}{}^{\prime }\left(kn_{h}R\right) & -P_{h}C_{l}{}^{\prime }\left(kn_{h}R\right) & 0 & 0 & 0\\ 0 & S_{l}\left(kn_{h}R_{h}\right) & C_{l}\left(kn_{h}R_{h}\right) & -S_{l}\left(kn_{c}R_{h}\right)\; & -C_{l}\left(kn_{c}R_{h}\right)\; & 0\\ 0 & P_{h}S_{l}{}^{\prime }\left(kn_{h}R_{h}\right) & P_{h}C_{l}{}^{\prime }\left(kn_{h}R_{h}\right) & -P_{c}S_{l}{}^{\prime }\left(kn_{c}R_{h}\right) & -P_{c}C_{l}{}^{\prime }\left(kn_{c}R_{h}\right) & 0\\ 0 & 0 & 0 & S_{l}\left(kn_{c}R_{hc}\right) & C_{l}\left(kn_{c}R_{hc}\right) & -C_{l}\left(kn_{0}R_{hc}\right)\\ 0 & 0 & 0 & P_{c}S_{l}{}^{\prime }\left(kn_{c}R_{hc}\right) & P_{c}S_{l}{}^{\prime }\left(kn_{c}R_{hc}\right) & -P_{0}C_{l}{}^{\prime }\left(kn_{0}R_{h}\right) \end{array}\right|=0$$

where *R_c_* = *R* + *d*, *R_h_* = *R* + *d_h_* and *R_hc_* = *R* + *d_h_* + *d*, *S_l_* and *C_l_* are the Ricatti–Bessel functions defined as: $S_{l}\left(x\right)=xj_{l}\left(x\right),\;C_{l}\left(x\right)=xy_{l}\left(x\right)$. In general, Equation (5) will have multiple solutions which we number using an integer index *p*. This makes each mode defined by three integers *p*, *l* and *m*. The three quantities *p*, *l-m* and *m* correspond to the number of nodes in the radial, azimuthal and polar directions, respectively. The modes themselves are frequency-degenerate in *m*. In all our calculations, for a given *p* we chose the value of *l* to correspond to the mode whose wavelength is the closest to 1550 nm, this being the widely used telecom wavelength. Once the eigen-wavelengths *λ* are obtained, it is fairly straightforward to obtain the sensing sensitivity by calculating the quantity $\partial \lambda /\partial n_{c}$. For any sensor, the highest possible sensitivity is desired. However, in the case of sensors that include porous coatings, the thickness of the coating needs to be also taken into consideration. The thicker the porous coating is, the more likely it is for the porous layer to deform, crack or even to collapse onto itself, which makes obtaining very thick porous coating very challenging. Therefore, the aim of our calculations will be to obtain the optimum parameters that achieve the highest sensitivity for lowest possible thickness of the porous coating. The parameters which we vary are *n_c_*, *R* and *d* for the single layers system, and are *n_c_*, *R*, *d*, *d_h_* and *n_h_* for the two-layer system. Since *d* is the parameter which is the easiest to vary experimentally, in the graphs we will always plot $\partial \lambda /\partial n_{c}$ in respect to *d* for select values of other parameters. Without loss of generality, the results for the TE modes are presented throughout the paper, while the TM modes are discussed only in chapter 3.3. In addition to $\partial \lambda /\partial n_{c}$, the effective refractive index *n_eff_* is also calculated for each mode. The *n_eff_* of a WGM mode is defined as [27]:

(6) $$n_{eff}=\frac{m\lambda }{2\pi \left(R+d+d_{h}\right)}$$

where |m|<l.

## 3. Results and Discussion

### 3.1. Microsphere Coated with a Single Layer

**Figure 2:**
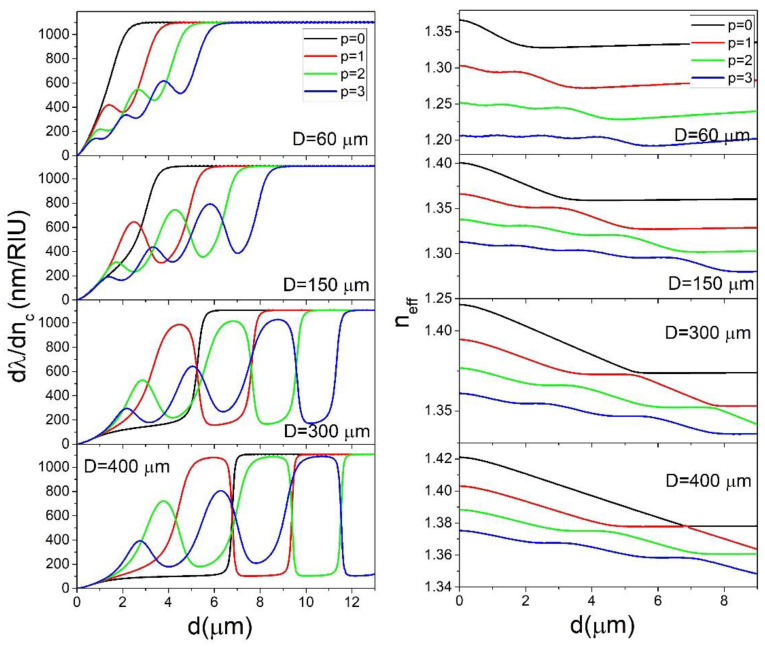
The dependence of $\partial \lambda /\partial n_{c}$ (**left**) and *n_eff_* for *l* = *m* (**right**) on *d* for *n_c_* = 1.4 calculated for select values of *p* and *D*.

**Figure 3:**
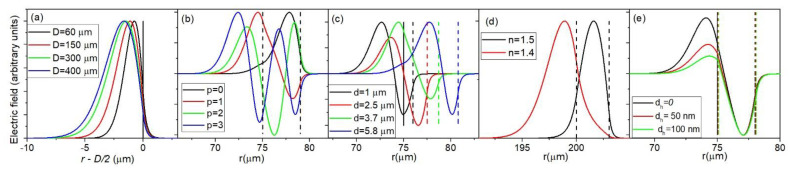
The normalized radial electric field profiles in respect to the distance from the center of the sphere *r* for: (**a**) *d* = 0, *p* = 0; (**b**) *D* = 150 μm, *d* = 4 μm; (**c**) *D* = 150 μm; *p* = 1; (**d**) D = 400 μm; *d* = 3 μm; *p* = 0; (**e**) *D* = 150 μm, *d* = 3 um, *p* = 1, *n_h_* = 1.9. The vertical lies correspond to the interfaces between the *n_s_*, *n_h_*, *n_c_* and *n*_0_ layer.

In Figure 2, the dependence of $\partial \lambda /\partial n_{c}$ on *d* is shown for *n_c_* = 1.4 which would correspond to a porous silica layer with a porosity of 9%, a reasonable value for sol-gel porous silica [13,14]. It is important to note that in this case *n_c_* < *n_s_* since in the opposite case very different behavior is observed, as will be discussed later. It is clearly visible that for a given sphere size, the modes with different values of *p* show different behavior. The sensitivity of the *p* = 0 mode increases with increasing *d* until reaching a maximum value of about 1100 nm/RIU. The thickness *d* for which the sensitivity reaches this maximum value is higher for larger spheres. For example, for a sphere 60 μm in diameter (*D* = 60 μm), 90% of the maximum value is reached for *d* = 2.1 μm, while for *D* = 400 μm it is reached for *d* = 6.9 μm. The reason for this effect is because the electric field of the WGMs for larger spheres extends further into the interior of the sphere than in the case of smaller spheres. This is a consequence of simple scaling: bigger spheres lead to a larger radial width of the modes. Therefore a larger *d* is needed to confine the whole electric field of the mode inside the coating. This is illustrated in Figure 3a, where the radial electric field profiles for the *p* = 0 mode are shown for different sphere diameters for uncoated spheres.

This effect has to be taken into account when designing a WGM gas sensor. When using a smaller sphere for gas sensor manufacturing, the applied coating does not need to be very thick to reach high sensitivities. This would greatly ease the manufacturing process, since thinner layers are less likely to crack and/or collapse during the coating process. Any cracking of the layer can decrease the Q-factor of the sphere. Although this would not decrease the sensitivity, it would decrease the smallest detectable shift of the mode frequency, which would in turn reduce the detection limit. On the other hand, the drawback of using smaller spheres is that they can be more difficult to produce. If the standard method for WGM microspheres production based on melting the tip of a telecom fiber is used, the production of spheres smaller than the diameter of the fiber (usually 125 μm) would have to include an additional tapering step. For the *p* = 1 modes, increasing *d* leads to an initial increase of $\;\partial \lambda /\partial n_{c}$ until *d* reaches a local maximum, upon which $\partial \lambda /\partial n_{c}$ tends to decrease before reaching a local minimum, after which it increases again until reaching the maximum value, which is about 1100 nm/RIU, the same as for the *p* = 0 mode. For the *p* = 2 modes the behavior is similar; the only difference is that the sensitivity passes through two local maxima before reaching its maximum value which is, again, 1100 nm/RIU. The reason the modes with different values of *p* show different behavior is because of the different spreading of the modes into the interior of the sphere. This is illustrated in Figure 3b for the particular case of *D* = 150 μm, *d* = 4 μm. The modes with different *p* have different electric fields profiles, the modes with higher *p* spreading deeper into the sphere. This means that in general, the modes with lower *p* will have higher sensitivities. This is generally the case; the modes with lower *p* always reach their maximum sensitivity for lower values of *d*. However, for particular values of *d* it is not always the case that lower *p* means higher sensitivity. It is the confinement of the WGM electric field in the coating that is the crucial parameter for determining sensitivity, and sometimes even if one WGM is spread deeper into the sphere than another WGM, it can have more of its electric field inside the coating. For example, in Figure 3b, the *p* = 2 mode reaches deeper into the sphere than the *p* = 1 mode, however two out of three of its lobes are entirely confined inside the coating, while regarding the two lobes of the *p* = 1 mode, the lobe inside the coating has much smaller intensity than the lobe outside the coating making $\partial \lambda /\partial n_{c}$ of the *p* = 2 mode higher than for the *p* = 1 mode, as can be seen from Figure 2. To further explain this effect we can take a look at Figure 3c, where the electric field profiles for the particular case of the *p* = 1 mode are shown for particular values of *d* that correspond to the local minima and maxima of $\partial \lambda /\partial n_{c}$. For *d* = 1 μm, the thickness is very low, so most of the electric field is located outside of the coating. For *d* = 2.5 μm we reach the first local maximum, where one lobe is located entirely inside the coating (exterior lobe) and one in the sphere core (interior lobe). Upon further increase of *d*, more of the interior lobe starts entering the coating, thus increasing the sensitivity, while simultaneously the peak intensity of the exterior lobe decreases in respect to the peak intensity of the interior lobe, thus decreasing the sensitivity. This results in the sensitivity first reaching a local minimum for *d* = 3.7 μm and then reaching its global maximum for *d* = 5.8 μm when the entire electric field of both lobes is confined inside the coating. It is interesting to note that the value of the first local maximum of $\partial \lambda /\partial n_{c}$ increases with increasing sphere diameter: while for a 60 μm sphere the first maximum is only 38% of the global maximum, for a 400 μm sphere it is as high as 97% of the global maximum. This means that for larger spheres, in practical applications, it is the *p* = 1 mode that is more suitable for sensing application instead of the *p* = 0 mode.

From Figure 2 we can also see that, at fixed *d*, the $\partial \lambda /\partial n_{c}$ can be very different for different values of *p*. This means that to construct sensors which would operate at maximum $\partial \lambda /\partial n_{c}$, selective coupling to modes with a particular *p* is required. This can be achieved by selectively coupling to modes with select values of *n_eff_* using an experimental coupling method which is selective to *n_eff_* (for example prism coupling). In general, according to Equation (6), the WGMs with the same *l* and *p* but with different *m* can have very different values of *n_eff_* ranging from the maximum for *l* = *m* to 0 for *m* = 0. In practice, however, the *m* = 0 modes are never observed, since their electric field extends significantly across the polar regions of the sphere, to one of which the stem that the sphere is attached to is located. The stem, which is used for manipulating the sphere, introduces significant losses to the WGM which degrades the Q-factor of the WGM enough to make it non-distinguishable in the experimental setup used for sensing. However, modes with *m* < *l* where *m* is not too low will be located mostly in the equatorial plane of the sphere, although they are slightly more delocalized than in the *m* = *l* case. In general, it will be very difficult to distinguish experimentally between, for example, the *m* = *l* and the *m* = *l −* 1 modes. In all subsequent Figures we always plot *n_eff_* for the *m* = *l* mode for a given *p*, although we have to always take into account that *n_eff_* can also be slightly smaller than for the *l* = *m* case. In Figure 2 (right), the calculated values of *n_eff_* are shown for the same system for which $\partial \lambda /\partial n_{c}$ is shown in Figure 2 (left). We can see that in general the modes with higher *p* have lower *n_eff_*. This means that the *p* = 0 mode can always be selectively coupled to if we were to couple using a *n_eff_* that is smaller than *n_eff_* for *p* = 0, *l* = *m* but larger than *p* = 1, *l* = *m*. This is fortunate, since for most spheres the *p* = 0 mode is the one that is the most useful for real-life sensing application. However, as already mentioned, it is the *p* = 1 mode that is better-suited for bigger spheres. For example, for *D* = 400 μm and *d* = 5.4 μm, the $\partial \lambda /\partial n_{c}\;$ is 1060 nm/RIU for the *p* = 1 mode and only 104 nm/RIU for the *p* = 0 mode. Unfortunately, for the same system according to Equation (6) the *p* = 1, *l* = *m* mode should have the same *n_eff_* as the *p* = 0 for *l* = 1156, *m*= 1148 which means that when targeting the *p* = 1 mode we could also couple to the *p* = 0 mode. In Figure 2 it is also noticeable that for smaller spheres the overall difference between *n_eff_* of modes with different *p* is much larger than for bigger spheres. For example, for *D* = 60 μm the *n_eff_* for the *p* = 0 and *p* = 1 modes can differ in magnitude up to 0.06 RIU while in the case of *D* = 400 μm this difference is less than 0.01 RIU. The higher this difference between *n_eff_* is, the smaller is the accuracy of the experimental setup’s selective part (such as angle of incidence onto the prism in the case of prism coupling) needed to distinguish the two modes. From all that was said above, we conclude that in the case of a single coated sphere with *n_c_* = 1.4, the smaller the diameter of the sphere is, the better suited the sphere is for sensing applications.

**Figure 4:**
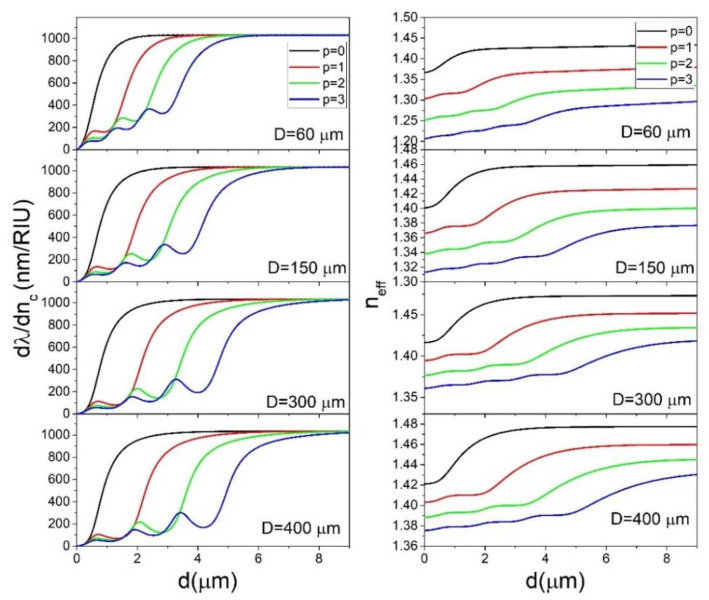
The dependence of $\partial \lambda /\partial n_{c}$ (**left**) and *n_eff_* for *l* = *m* (**right**) on *d* for *n_c_* = 1.5 calculated for select values of *p* and *D*.

In Figure 4 the results of calculations of $\partial \lambda /\partial n_{c}$ for *n_c_* = 1.5 are shown. Note that in this case, *n_c_* > *n_s_*. We see that the results are vastly different than in the case of *n_c_* < *n_s_*. While for *n_c_* = 1.4 the spheres with different diameters showed very different behavior, for *n_c_* = 1.5 the $\partial \lambda /\partial n_{c}$ dependence on *d* is almost identical for different sphere diameters. The value of *d* needed to reach the maximum sensitivity is still increasing with *D*, however this increase is much smaller than in the *n_c_* = 1.4 case. The maximum value of the sensitivity is 1000 nm/RIU which is a bit smaller than in the *n_c_* = 1.4 case. Additionally, the values of the local maxima for *p* > 0 modes are much smaller than in the case of *n_c_* = 1.4. This means that, for all practical purposes, for *n_c_* > *n_s_* it is the *p* = 0 mode that should be targeted for sensor production. Additionally, the *n_eff_* difference between the *p* = 0 and *p* = 1 modes is much larger than in the *n_c_* = 1.4 case, ranging from 0.08 RIU for *D* = 60 μm to 0.05 RIU for *D* = 200 μm. We can conclude that in the *n_c_* = 1.5 case, the coating thickness required to produce an efficient sensor is much smaller than in the *n_c_* = 1.4 case. Additionally, while the sphere diameter is a very important parameter for sensor design in the *n_c_* = 1.4 case, in the *n_c_* = 1.5 case different sphere diameters all produce similar results, which significantly simplifies the sensor design since the sphere diameter used can vary in a wide range. The reason behind the vastly different behavior in the two presented cases is the fact that in the *n_c_* >*n_s_* case the coating can serve as a wave-guiding structure, while in the *n_c_* < *n_s_* case the coating serves as a cladding for the mode confined in the sphere interior. If *n_c_* > *n_s_*, the coating is sandwiched between two lower refractive index layers, meaning that it can serve as a waveguide if *d* is thick enough to support a wave-guiding mode. The thickness *d* required to support a particular mode depends only on the refractive indices *n_c_*, *n_s_* and *n*_0_ and not on the sphere diameter. Because of this, the graphs in Figure 3 all show similar behavior, regardless of sphere diameter. In the *n_c_* < *n_s_* case, the coating layer serves as a cladding for the mode located in the sphere interior meaning that the mode cannot be confined inside the coating. The radial spread of the mode will therefore be primarily determined by the spread of the mode in the sphere interior which is, as was already shown in Figure 3a, strongly dependent on the sphere diameter. This is illustrated in Figure 3d, where the electric field profiles are presented for the particular case *D* = 150 μm, *d* = 3 μm. While for the *n_c_* = 1.5 case the mode is completely confined inside the coating, in the *n_c_* = 1.4 case the mode spreads far into the interior in the sphere in a similar manner, as if the sphere was not coated at all.

**Figure 5:**
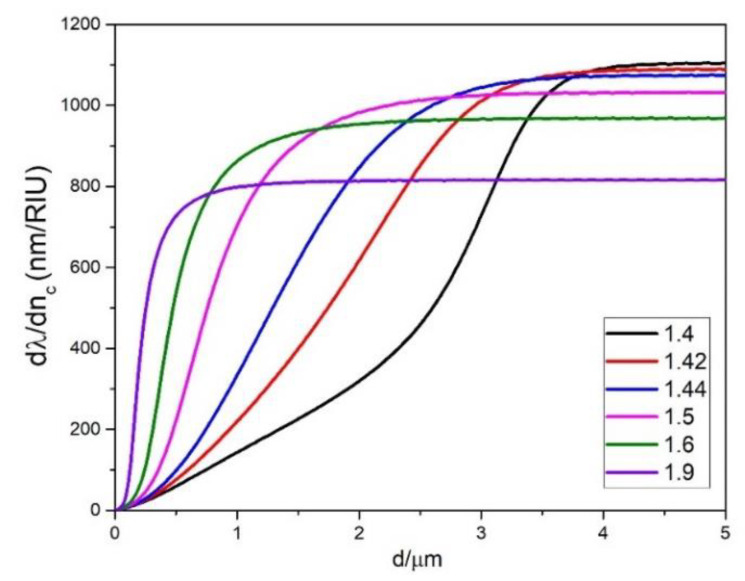
The dependence of $\partial \lambda /\partial n_{c}$ on *d* for the *p* = 0 mode for selected values of *n_c_* for *D* = 150 μm.

It is interesting to compare the $\partial \lambda /\partial n_{c}$ dependence on *d* for *p* = 0 and fixed *D* for different values of *n_c_* as is shown in Figure 5 for *D* = 150 μm. We can see that with increasing *n_c_* the maximum sensitivity decreases, although the value of *d* needed to reach the maximum is smaller. For example, for *n_c_* = 1.4, the maximum of $\partial \lambda /\partial n_{c}$ is 1100 nm/RIU for *d* > 4 μm, while for *n_c_* = 1.9 it is 800 nm/RIU for *d* = 1 μm. For practical applications, this four-fold decrease in the operating thickness might be more important than the 20% decrease of sensitivity. Producing sensors with slightly smaller sensitivity but with coating thicknesses which are much easier to produce experimentally can be a reasonable compromise.

### 3.2. Microsphere Coated with Two Layers

**Figure 6:**
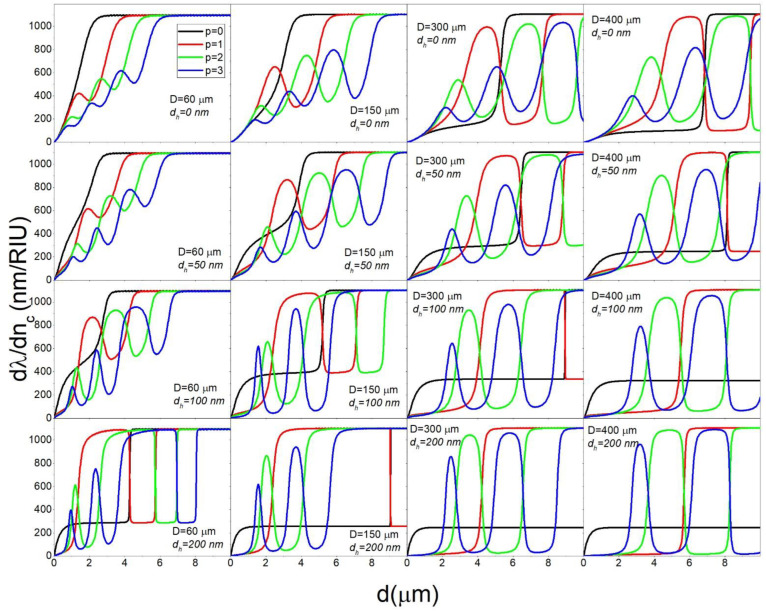
The dependence of $\partial \lambda /\partial n_{c}$ on *d* for *n_c_* = 1.4 and *n_h_* = 1.9 calculated for select values of *p*, *d_h_* and *D*.

In the previous section, it was shown that coating the microsphere with a low refractive index layer can result in very high sensitivities while at the same time requiring very thick coatings, especially for spheres with large diameters. To tailor the electric field of a confined mode, a thin high refractive index layer can be introduced next to the guiding layer. Even a very thin layer can be used to push out the electric field of a given mode outside the structure in which the mode is confined, depending on the geometrical parameters of the system in question. In our case, we can introduce a high refractive index layer (*n_h_*) between the sphere and the sensing layer, as shown in Figure 1b. In Figure 6, the calculated $\partial \lambda /\partial n_{c}$ in dependence on *d* for *n_s_* = 1.44, *n_c_* = 1.4, *n_h_* = 1.9 are shown for different values of *d_h_* and *D*.

We can see that, for the *p* = 0 mode, $\partial \lambda /\partial n_{c}$ increases due to the additional layer only for very small values of *d*. For example, for *d* = 1 μm and *D* = 150 μm an additional layer as thin as *d_h_* = 100 nm increases $\partial \lambda /\partial n_{c}$ from 143 to 321 nm/RIU. However, in the range in which this increase happens, $\partial \lambda /\partial n_{c}$ is always below the maximum value that is 1100 nm/RIU. On the other hand, the additional layer causes a significant increase of the value of *d* needed to reach the maximum of $\partial \lambda /\partial n_{c}$. For *p* > 0 modes, the overall effect of the added layer is more complicated. The most important conclusion that can be seen from Figure 6 for the *p* > 0 modes is that the additional layer can greatly increase the $\partial \lambda /\partial n_{c}$ at its local maxima. For example, when using a thin additional layer of *d_h_* = 100 nm the $\partial \lambda /\partial n_{c}$ for the *p* = 1 mode of a *D* = 150 μm sphere can reach 1000 nm/RIU for *d* = 3 μm, even though without the additional layer its first local maximum would only reach 644nm /RIU. For *p* > 1 modes this effect is also present, as summarized in Table 1. We can see that the additional high refractive index layer can be used to decrease the thickness *d* needed to achieve high values of $\partial \lambda /\partial n_{c}$, provided that we manage to selectively couple to higher order modes.

The reason that the high refractive layer has such a large influence on $\partial \lambda /\partial n_{c}$ is illustrated in Figure 3e for the particular case of *D* = 150 μm, *d* = 3 μm, *p* = 1. In this case the *p* = 1 mode has two lobes: the exterior lobe located in the coating and the interior lobe in the sphere core. The relative intensity of these two lobes is primarily dependent on the refractive indices on the interface between the coating and the sphere which are greatly modified by the addition of the thin high refractive layer between the sphere and the coating. This is evident in Figure 3e where the increase of *d_h_* leads to a significant increase of the intensity of the exterior lobe in respect to the interior lobe.

**Figure 7:**
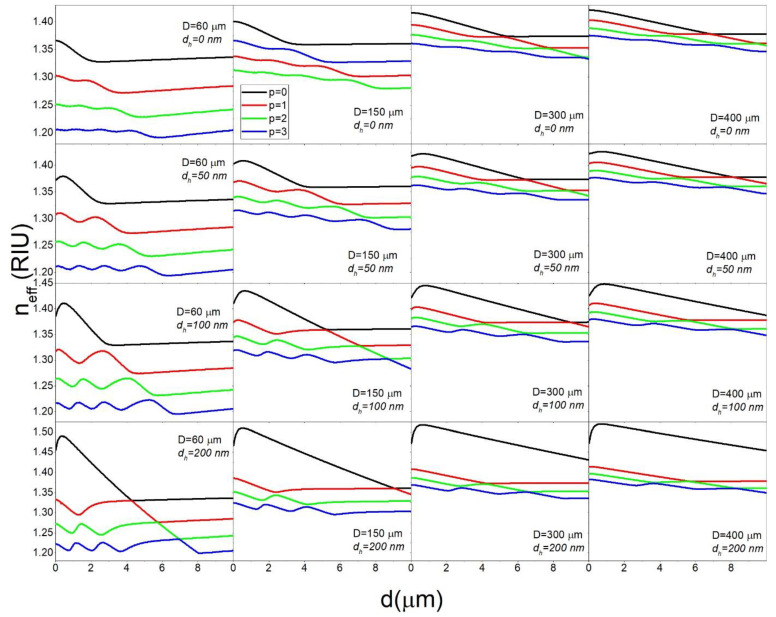
The dependence of *n_eff_* on *d* for *n_c_* = 1.4 and *n_h_* = 1.9 calculated for select values of *p*, *d_h_* and *D*.

**Figure 8:**
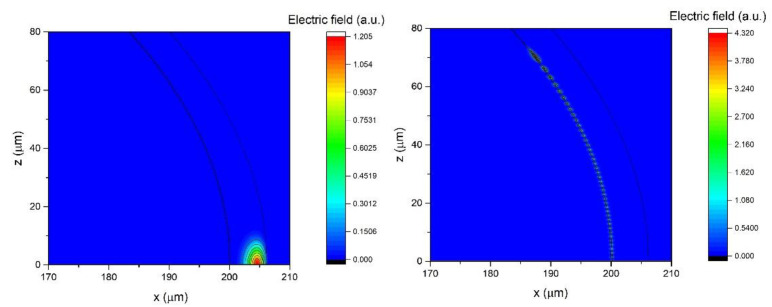
The electric field profiles for a 400 μm sphere with *d_h_* = 200 nm and *d* = 6 μm for the *p* = 1, *l* = *m* = 1156 mode (**left**) and the *p* = 0, *l* = 1239, *m* = 1156 mode (**right**). The origin *x* = *z* = 0 is in the center of the sphere. The *z* = 0 plane is the equatorial plane. The full lines represent boundaries between different layers.

The *n_eff_* calculated for the same parameters for which $\partial \lambda /\partial n_{c}$ were presented in Figure 6 are shown in Figure 7. We can see that the main effect of the additional high refractive index layer is to greatly increase the difference in *n_eff_* between the *p* = 0 and *p* > 0 modes. For example, while for *D* = 400 μm the difference between the *n_eff_* for the *p* = 0 and *p* = 1 modes is only about 0.01 RIU, with the addition of a 200 nm high refractive index layer this difference can be as high as 0.11 RIU. This would mean that the *p* = 1, *l* = 1156, *m* = 1156 and the *p* = 0, *l* = 1239, *m* = 1156 modes have the same *n_eff_*. In this case the *l*-*m* difference for the *p* = 0 mode is 83, which means that the mode is significantly delocalized towards the polar regions of the sphere. This is illustrated in Figure 8 where we can see that while the *p* = 1, *l* = 1156, *m* = 1156 mode is located at the equatorial plane of the sphere (within 10 μm of the equator), the *p* = 0, *l* = 1239, *m* = 1156 mode is distributed across the sphere up to 70 μm away from the equator. By choosing the correct design of the sphere and of the stem, the very delocalized *p* = 0 mode can be made to be so lossy as to effectively cease to exist. In this manner, the *p* = 1 mode can be selectively coupled to by choosing the *n_eff_* of the coupler to correspond to the *n_eff_* of the *p* = 1 *l* = *m* mode. By coupling to the *p* = 1 mode we can obtain maximum $\partial \lambda /\partial n_{c}$ using *d_h_* = 200 nm and *d* = 6 μm, while for the *p* = 0 we would need *d* = 7.25 μm to reach the maximum $\partial \lambda /\partial n_{c}$. Unfortunately, Figure 7 also shows that the *n_eff_* of all the *p* > 0 modes in the *d_h_* = 0–200 nm range tend to have very similar magnitudes which would make it very difficult to selectively couple to the *p* > 1 modes. While the *p* = 2,3… modes could offer a further decrease of the thickness needed to reach high $\partial \lambda /\partial n_{c}$, it could prove very challenging to selectively couple to them.

**Figure 9:**
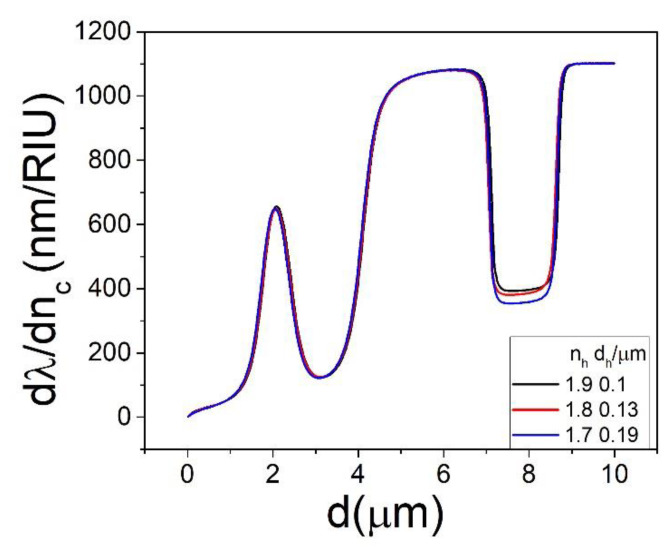
The dependence of $\partial \lambda /\partial n_{c}$ on *d* for the *p* = 2 mode for *n_c_* = 1.4 and *D* = 150 μm calculated for select values of *d_h_* and *n_h_*.

As expected, if we were to engineer a particular system with a pair of values of *d_h_* and *n_h_*, we can obtain a similar system which has a smaller *d_h_* and a larger *n_h_*, or vice versa. Changing *n_h_* has the effect of changing both the optical path length inside the layer and the Fresnel coefficients of refraction at the *n_s_*- > *n_h_* and *n_h_*- > *n_c_* boundaries, but the predominant effect is the optical path length change. This is illustrated in Figure 9 where $\partial \lambda /\partial n_{c}$ is found to be almost the same when calculated for three different pairs of *d_h_*, *n_h_* for a particular mode. Therefore, a sensor whose response is tailored to operate at certain values of *d_h_*, *n_h_* can be easily designed with the same sensitivity using a material with a different *n_h_* by simply modifying *d_h_*.

### 3.3. Comparison of the TE and TM Modes

**Figure 10:**
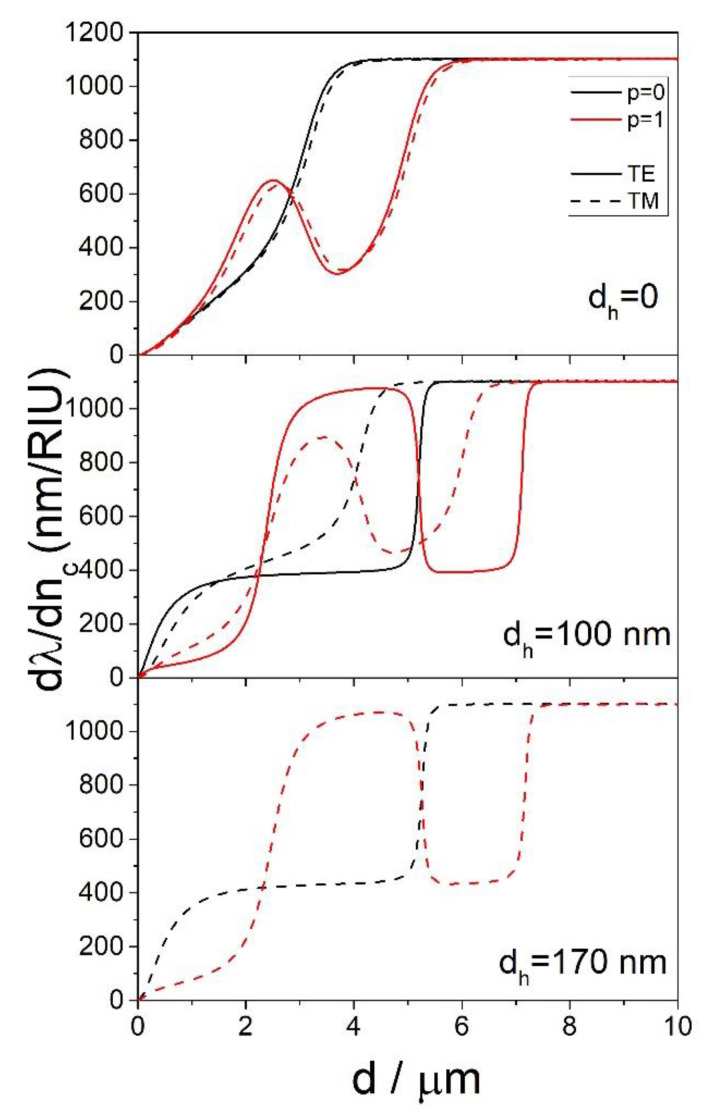
The dependence of $\partial \lambda /\partial n_{c}$ on *d* for *n_c_* = 1.4, *n_h_* = 1.9 and *D* = 150 μm for the TE and TM modes calculated for select values of *p* and *d_h_*.

The same calculations that were made for the TE modes have also been carried out for the TM modes. In the case of the sphere coated with one layer, the calculated $\partial \lambda /\partial n_{c}$ for the TE and TM modes are almost identical, as shown in Figure 10. In the case of the sphere coated with two layers, the TM modes show qualitatively the same dependence of $\partial \lambda /\partial n_{c}$ on *d* as the TE modes; the only difference is in the value of *d_h_* needed to achieve the same behavior. For example, from Figure 10 it is visible that for *d_h_* = 100 nm, the TE and TM modes have very different $\partial \lambda /\partial n_{c}$. However, the calculated $\partial \lambda /\partial n_{c}\;$ for the TE mode at *d_h_* = 100 nm is almost identical as for the TM mode and *d_h_* = 170 nm. This means that for practical applications, the TE modes are the ones that should be targeted, since they can be engineered to have the same $\partial \lambda /\partial n_{c}$ as the TM modes but for smaller values of *d_h_*.

## 4. Conclusions

We have presented a theoretical analysis of a sensing system based on a microsphere coated with a porous sensing layer. We have found that the fact whether the refractive index of the sphere *n_s_* is greater or smaller than the refractive index of the coating *n_c_* greatly affects the sensing sensitivity. If *n_c_ < n_s_*, the thickness needed to reach the optimum sensitivity depends significantly on sphere diameter, making smaller spheres the preferred option for sensor construction. On the other hand, for *n_c_ > n_s_*, the diameter of the sphere is not important, since it does not influence the overall sensitivity. We have found that, in general, the *p* = 0 mode is the most suitable one for practical sensing application, although for larger spheres (*D* > 400 μm) with *n_c_* < *n_s_*, the *p* = 1 mode is the more suitable one. In this case, however, the problem of selectively coupling to the *p* = 1 modes needs to be solved. By introducing an additional high-refractive index layer (as thin as 200 nm for *n_h_* = 1.9) the *p* = 1 mode can be engineered to have both an increased sensing sensitivity and an increased *n_eff_* mismatch with the *p* = 0 mode, which could facilitate selective coupling to the *p* = 1 mode.

## Figures and Tables

**Table 1:** Calculated $\partial \lambda /\partial n_{c}$ for *n_c_* = 1.4, *n_h_* = 1.9, *D* = 150 μm at *d* corresponding to the first local maximum for a given *p* for *d_h_* = 0 and for the optimum *d_h_* that maximizes $\partial \lambda /\partial n_{c}$ at *d*.

*p*	0	1		2		3		4	
*d* (μm)	4.1	3		2		1.56		1.31	
*d_h_* (nm)	0	0	100	0	200	0	220	0	220
$\partial \lambda /\partial n_{c}$ (nm/RIU)	1100	644	1000	312	864	186	640	134	460
